# Body Esteem among Tunisian adolescents: Prevalence and associated factors

**DOI:** 10.1192/j.eurpsy.2025.1112

**Published:** 2025-08-26

**Authors:** N. E. Ayadi, S. Bourgou, A. Ben Hamouda, F. Charfi, A. Ghenimi

**Affiliations:** 1 child and adolescent psychiatry department, University hospital Mongi Slim; 2 child and adolescent psychiatry department, Mongi Slim university Hospital, Tunis, Tunisia

## Abstract

**Introduction:**

Body esteem refers to an individual’s self-evaluation of their own body or appearance. It plays a critical role in the psychosocial development of adolescents, who become increasingly aware of their bodies and how they are perceived by others.

**Objectives:**

This study aims to examine the specificity of body esteem among Tunisian adolescents and its associated factors.

**Methods:**

This cross-sectional study was conducted via a survey among adolescents attending middle and high schools during the 2023-2024 school year. Participants provided written consent and completed a demographic information sheet, the Body Esteem Scale for Adolescents and Adults (BESAA), and the Adverse Childhood Experiences-International Questionnaire (ACE-IQ).

**Results:**

The study population consisted of 1,005 adolescents, with a sex ratio of 0.73 and a mean age of 14.62 years. We found that 88,1% of adolescents had high body esteem while 11,9% had low body esteem. We found that body esteem is statistically correlated with : low socioeconomic status (p=0,033, OR=0,58 (95%CI [0,35-0,94])), with the history of organix disease (p<0,001, OR=0,415 (95%[0,265-0,650])), with BMI measurements (p<0,001) specifically with obesity (p=0,014, OR=0,497 (95%CI [0,292-0,846])). The findings suggested that selfie practice was significantly associated with body esteem (p=0,009, OR=1,819 (95% [1,18-2,8])) and that selfie posting on social media was statistically correlated to appearance esteem *with* p-value =0,027.

Additionally, we found that body esteem was directly and significantly associated with all the adverse childhood experiences except for collective violence. Detailed results are shown in the table below:

**Table:** 

Adverse childhood experiences		Family relations	Neglect	Family dysfunction	Psychological abuse	Physical abuse	Sexual abuse	Peer violence	Community violence
**Body Esteem**	***p***	0,018	<0,001	<0,001	0,001	<0,001	0,001	<0,001	0,007
	**OR (95% CI)**	0,55 (0,34-0,89)	0,33 (0,22-0,5)	0,39 (0,26-0,6)	0,496 (0,32-0,77)	0,429 (0,28-0,64)	0,47 (0,3-0,73)	0,43 (0,27-0,7)	0,53 (0,33-0,84)

**Conclusions:**

By understanding the associated factors of body esteem, this study contributes to a broader comprehension of adolescents’ well-being and offers insights for creating a safer and more supportive environment for young people in our country.

**Disclosure of Interest:**

None Declared

